# Similar Outcomes in Minimally Invasive versus Open Management of Primary Pancreatic Neuroendocrine Tumors: A Regional, Multi-Institutional Collaborative Analysis

**DOI:** 10.3390/cancers14061387

**Published:** 2022-03-09

**Authors:** Thomas L. Sutton, Rodney F. Pommier, Skye C. Mayo, Erin W. Gilbert, Pavlos Papavasiliou, Michele Babicky, Jon Gerry, Brett C. Sheppard, Patrick J. Worth

**Affiliations:** 1 Department of Surgery, Division of General Surgery, Oregon Heath & Science University (OHSU), Portland, OR 97239, USA; suttoth@ohsu.edu (T.L.S.); gilberte@ohsu.edu (E.W.G.); sheppard@ohsu.edu (B.C.S.); 2 Department of Surgery, Division of Surgical Oncology, Knight Cancer Institute, Oregon Heath & Science University (OHSU), Portland, OR 97239, USA; pommierr@ohsu.edu (R.F.P.); mayos@ohsu.edu (S.C.M.); 3 Northwest Permanente, P.C., Portland, OR 97232, USA; pavlos.papavasiliou@kp.org; 4 The Oregon Clinic, Center for Advanced Surgery, Portland, OR 97213, USA; mbabicky@orclinic.com (M.B.); jgerry@orclinic.com (J.G.)

**Keywords:** pancreatic neuroendocrine tumor, minimally invasive surgery, oncologic outcomes, perioperative outcomes

## Abstract

**Simple Summary:**

Pancreatic neuroendocrine tumors (PNETs) are rare tumors of the pancreas that are often curable with surgery. Due to their rarity, it is difficult to study whether newer techniques for removing PNETs, specifically minimally invasive surgeries, are as safe and effective as open surgeries in these patients. In this study, we pooled the experience of multiple high-volume institutions who remove PNETs, and studied outcomes in open and minimally invasive surgeries. We discovered that patients receiving a minimally invasive surgery were equally likely to remain alive and without disease as patients receiving an open surgery. Additionally, there was no difference in the most common complications experienced by patients receiving these operations. Therefore, we can recommend the routine use of minimally invasive surgery techniques in appropriately selected patients with PNET, if offered by their surgeon.

**Abstract:**

In pancreatic neuroendocrine tumors (PNETs), the impact of minimally invasive (MI) versus open resection on outcomes remains poorly studied. We queried a multi-institutional pancreatic cancer registry for patients with resected non-metastatic PNET from 1996–2020. Recurrence-free (RFS), disease-specific survival (DSS), and operative complications were evaluated. Two hundred and eighty-two patients were identified. Operations were open in 139 (49%) and MI in 143 (51%). Pancreaticoduodenectomy was performed in 77 (27%, *n* = 23 MI), distal pancreatectomy in 184 (65%, *n* = 109 MI), enucleation in 13 (5%), and total pancreatectomy in eight (3%). Median follow-up was 50 months. Thirty-six recurrences and 13 deaths from recurrent disease yielded 5-year RFS and DSS of 85% and 95%, respectively. On multivariable analysis, grade 1 (HR 0.07, *p* < 0.001) and grade 2 (HR 0.20, *p* = 0.002) tumors were associated with improved RFS, while T3/T4 tumors were associated with worse RFS (OR 2.78, *p* = 0.04). MI resection was not associated with RFS (HR 0.53, *p* = 0.14). There was insufficient mortality to evaluate DSS with multivariable analysis. Of 159 patients with available NSQIP data, incisional surgical site infections (SSIs), organ space SSIs, Grade B/C pancreatic fistulas, reoperations, and need for percutaneous drainage did not differ by operative approach (all *p* > 0.2). Nodal harvest was similar for MI versus open distal pancreatectomies (*p* = 0.16) and pancreaticoduodenectomies (*p* = 0.28). Minimally invasive surgical management of PNETs is equivalent for oncologic and postoperative outcomes.

## 1. Introduction

Pancreatic neuroendocrine tumors (PNET) represent a relatively rare tumor with unique oncologic behavior, especially when compared to other tumors arising in the pancreas [1]. Much has evolved regarding their detection and management in the last decade, with novel imaging strategies, therapeutics, and classification schema. There has also been a shift in the indications for and approaches to surgical resection of these tumors. Laparoscopic pancreatectomies were first described in the early 1990s, confined largely to distal pancreatectomy, with the first reported robotic distal pancreatectomy (a PNET) in 2003 [2,3,4,5]. Comfort with these minimally invasive pancreatectomies has increased, and these procedures are frequently being performed via laparoscopic or robotic approaches, including head of pancreas tumors, which are more difficult to resect minimally invasively [6]. As with any shift in technique, head-to-head prospective comparison is confounded by training, experience, improvements in technologies, and evolving indications. To date, one trial comparing minimally invasive to open distal pancreatectomy has provided randomized, prospective insight into differences in surgical approach [7]. The remainder of the literature is confined to retrospective review, which is frequently single-center data, highly biased by individual surgeon choices, or multi-center collaboratives, typically comprised of large, tertiary referral centers. The Greater Portland Pancreatic Cancer Registry is a regional database comprised of a diversity of surgeons, hospital settings, payor mixes, and referral patterns. Herein we sought to characterize changes in approach to primary, non-metastatic pancreatic neuroendocrine tumors over time, and to evaluate outcomes using a large regional database of several high-volume pancreatic referral centers with extensive minimally invasive experience. 

## 2. Materials and Methods

### 2.1. Patient Identification and Data Source

Patients with nonmetastatic PNET undergoing curative-intent resection from 1996–2020 were identified from the Greater Portland Pancreatic Cancer Registry, which is composed of cancer registry data from four American College of Surgeons’ Commission on Cancer (CoC)-accredited health systems, formatted according to North American Associated for Central Cancer Registries (NAACCR) standards. Our registry captures standard NAACCR variables (Oregon Health and Science University (OHSU), Providence Health System, Kaiser Permanente Northwest, and Legacy Health) [8]. Matched perioperative data through the National Surgical Quality Improvement Project (NSQIP), including pancreatectomy procedure-targeted variables, were also retrieved from two institutions, who captured 100% of pancreatectomy procedures (OHSU and Providence Portland Medical Center) [9]. Patients were separated into groups by open versus minimally invasive (i.e., either laparoscopic or robotic) surgical approach. Patients for whom the surgical approach could not be identified were excluded from the study (*n* = 20). 

Prior to 2010, the AJCC 6th edition staging system for pancreatic malignancies was utilized to assign tumor grade to patients [10]; following 2010, PNET grading was defined by WHO definitions utilizing mitotic count and Ki67 thresholds [11]. For analyses that included tumor grade as a predictive variable (e.g., for RFS and DSS), patients were analyzed both including and excluding the pre-2010 cohort with no significant effect on the ultimate analysis outcome. For remaining tumor features, all staging was updated to reflect the AJCC 8th edition guidelines. There were no patients in the present cohort who were well differentiated with grade 3 disease per updated 2017 WHO guidelines [12]; therefore, tumor differentiation was reported but not utilized as a variable in the analysis of oncologic outcomes.

### 2.2. Outcomes

The primary outcomes of interest were recurrence-free survival (RFS), measured from the date of resection, and disease-specific survival (DSS), defined as time from diagnosis to death in the presence of recurrent disease, exclusive of death without evidence of disease. Due to limitations of the dataset, exact cause of death could not be determined, preventing more specific definitions of DSS.

Secondary outcomes of interest included surgical margins and nodal harvest. Additionally, incisional surgical site infections (SSIs), organ space SSIs, postoperative pancreatic fistulas (POPFs) inclusive of biochemical leaks, and clinically relevant POPFs (crPOPFs), need for perioperative blood transfusion, and readmission within 30 days were assessed for patients with matched NSQIP outcomes data. POPF-related outcomes were defined per the International Study Group of Pancreatic Fistula (ISGPF) 2016 guidelines. PMID: 28040257

### 2.3. Statistical Analysis

Clinicopathologic data were evaluated for procedure groups, reporting categorical variables as numbers and percentages and reporting numerical variables as medians with interquartile ranges (IQR). Fisher’s exact test, Chi-squared testing, and independent samples t-testing were utilized as appropriate. Analysis of RFS and DSS were performed using Kaplan–Meier analysis with log-rank testing, and Cox proportional hazards modeling following verification of the proportional hazard assumption. Binary postoperative outcomes (e.g., SSIs) were analyzed with logistic regression. Operative approach was analyzed in an intention-to-treat fashion unless otherwise specified (i.e., all conversions to open were analyzed with the minimally invasive group).

All multivariable analysis was performed using single backward elimination using likelihood ratios of variables with univariable *p* < 0.2, stopping when further elimination would reduce model fit with *p* < 0.05. Due to insufficient events of interest, multivariable analysis was not performed for DSS. For oncologic outcomes, final models were derived from the cohort at large. Independently significant variables were then applied to modeling outcomes of subgroups (e.g., patients undergoing distal pancreatectomy), adding operative approach to the model if not independently significant in the larger cohort.

All methods were pre-specified, and all tests are two-sided. For analysis, missing data for continuous variables were assigned the mean value of the relevant operative approach subgroup (e.g., for length of stay or operative duration); patients missing data for categorical variables were assigned to a separate dummy variable. Statistics were performed in SPSS 26 (IBM Corp., Armonk, NY, USA). Figures were created in Prism 9.0.2 (GraphPad Software, San Diego, CA, USA).

## 3. Results

### 3.1. Clinicopathologic Characteristics

Two hundred and eighty-two patients with resected nonmetastatic PNET were identified (Table 1). Operations were open in 139 (49%) and MI in 143 (51%). Pancreaticoduodenectomy was performed in 77 (27%, *n* = 23 MI), distal pancreatectomy in 184 (65%, *n* = 109 MI), enucleation in 13 (5%), and total pancreatectomy in eight (3%). NSQIP data were available in 159 patients (*n* = 92 minimally invasive, *n* = 67 open), and procedure-targeted data for pancreatectomies were available in 128 (*n* = 83 minimally invasive, *n* = 45 open). There were no significant demographic-level differences by operative approach, however, significant differences existed for operation/tumor location, tumor size, and AJCC stage, with patients undergoing open resection more likely to have larger more advanced tumors in the pancreatic head than patients undergoing minimally invasive resection.

**Figure 1:**
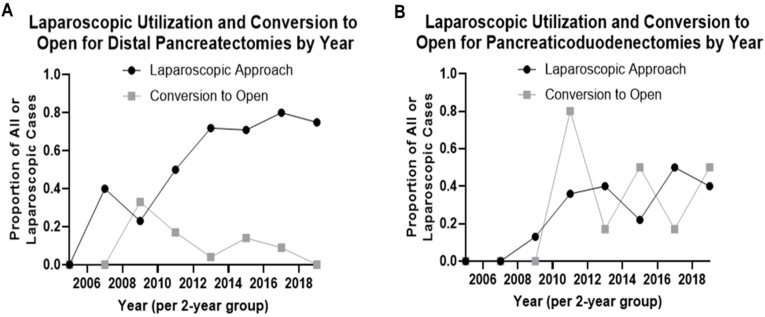
Temporal trends in attempted minimally invasive resections and proportion of attempted minimally invasive resections converted to open in patients with pancreatic neuroendocrine tumors. Trends in minimally invasive approach shown for (**A**) distal pancreatectomy and (**B**) pancreaticoduodenectomy. Data shown as 2-year bins starting in 2005, before which there were no laparoscopic resections attempted for either procedure.

Fifteen percent (*n* = 21) of operations starting with a minimally invasive approach had unplanned conversion to open, with significant differences by procedure type; unplanned conversion to open occurred in 10 of 24 pancreaticoduodenectomies/total pancreatectomies (42%) compared to 11 of 109 distal resections (10.1%, *p* < 0.001). None of the 10 enucleations were converted to open. Utilization of minimally invasive approaches increased for both distal pancreatectomies and pancreaticoduodenectomies starting in 2007 (Figure 1).

### 3.2. Primary Outcomes: Recurrence-Free and Disease-Specific Survival

**Figure 2:**
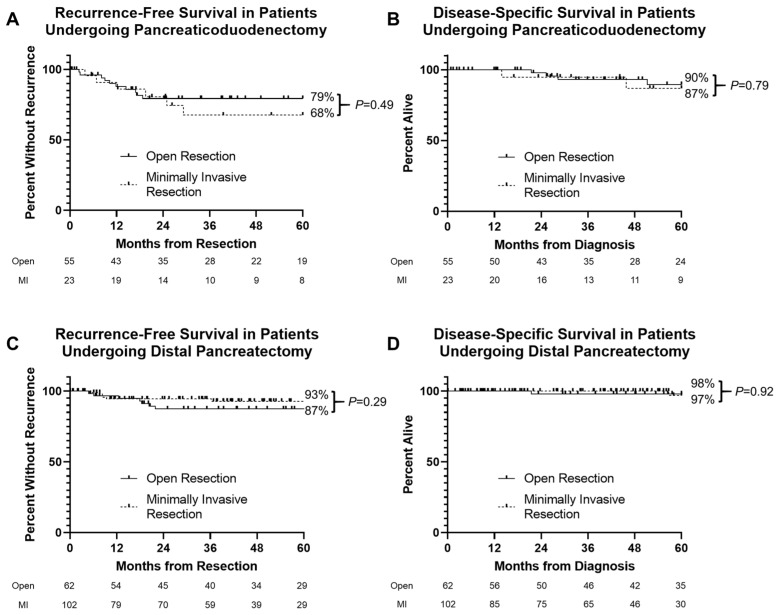
Kaplan–Meier plot of recurrence-free (RFS) and disease-specific survival (DSS) in patients undergoing resection of pancreatic neuroendocrine tumors. (**A**,**C**) 5-year RFS was not significantly different by operative approach for patients undergoing pancreaticoduodenectomy (log rank *p =* 0.49) and distal pancreatectomy (log rank *p =* 0.29). (**B**,**D**) 5-year DSS was not significantly different by operative approach for patients undergoing pancreaticoduodenectomy (log rank *p =* 0.79) and distal pancreatectomy (log rank *p =* 0.92).

Oncologic follow-up past 30 days from operation was available for 261 patients, including 165 distal pancreas resections and 78 pancreaticoduodenectomies. Median follow-up was 50 months from diagnosis (range 1–240 months). Thirty-six recurrences and 13 deaths from recurrent disease yielded 5-year RFS of 85% and 5-year DSS of 95% for the entire cohort. On Kaplan–Meier analysis, in patients undergoing pancreaticoduodenectomy, 5-year RFS was 79% versus 68% for patients with open and minimally invasive resections, respectively (Figure 2A, log rank *p* = 0.49); 5-year DSS was 90% and 87%, respectively (Figure 2B, log rank *p =* 0.79). In patients undergoing distal pancreatectomy, 5-year RFS was 93% versus 87% in patients with minimally invasive versus open resections, respectively (Figure 2C, log rank *p* = 0.29), while 5-year DSS was 98% and 97%, respectively (Figure 2D, log rank *p* = 0.79). There was no significant difference in survival with operative approach when stratifying by AJCC stage.

On univariable analysis of 261 patients with oncologic follow-up, factors associated with worse 5-year RFS included LVI, T3/T4 tumors, node positivity, and R1 margins (Table 2). Compared to grade 3 tumors, grade 2 and grade one tumors have improved 5-year RFS. A minimally invasive approach was not associated with 5-year RFS, although met the threshold for inclusion in the initial multivariable model; operative approach was removed from the final model during single backwards elimination. On multivariable analysis, tumor T stage and tumor grade were independently associated with 5-year RFS, while an R1 margin remained in the final model but were not statistically significant.

Excluding patients with total pancreatectomies/enucleations and stratifying for operation type, a minimally invasive approach was not independently associated with different 5-year RFS when added to the final multivariable model, either when analyzed per intention-to-treat (HR 0.93, 95% CI 0.42–2.06, *p* = 0.86) or ultimate operative approach (HR 0.69, 95% CI 0.28–1.70, *p* = 0.42).

On univariable analysis, older age, LVI, and T3/T4 tumors were associated with worse 5-year DSS, while grade 1 or 2 tumors were associated with improved 5-year DSS (Table 3). There was insufficient mortality in the setting of recurrent disease to evaluate DSS with multivariable analysis.

### 3.3. Secondary Outcome: Patterns of Recurrence

**Figure 3:**
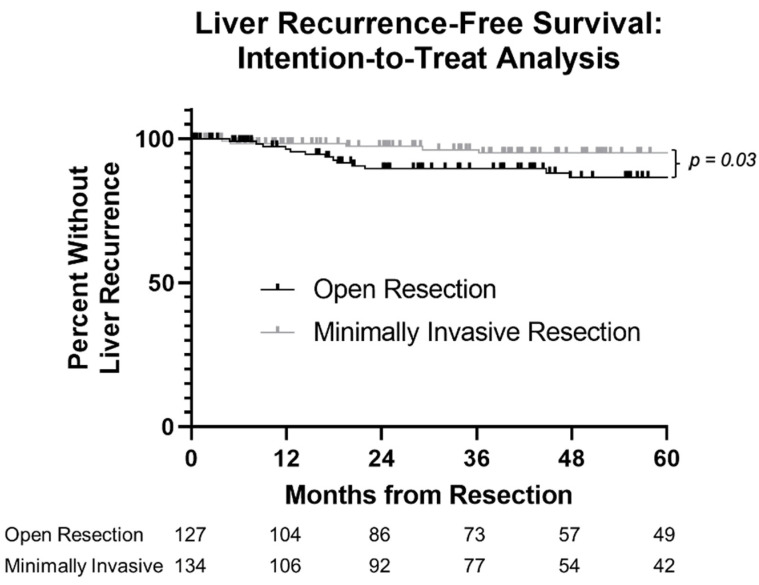
Kaplan–Meier plot of liver recurrence-free survival in patients undergoing resection of pancreatic neuroendocrine tumors, stratified by operative approach. Five-year liver-RFS 87% versus 95%, log-rank *p* = 0.03.

Of 36 recurrences, 13 (36%) were locoregional, one (3%) was peritoneal, one (3%) was lung-only, three (8%) were lung and liver, and 18 (50%) were liver-only. Open resection was associated with worse liver-RFS on Kaplan–Meier analysis (5-year liver-RFS 87% versus 95%, log-rank *p =* 0.03, Figure 3). This association of minimally invasive approach with improved liver-RFS did not persist when accounting for the AJCC pathologic stage (HR 0.50, 95% CI 0.18–1.40, *p* = 0.19) or the presence of lymphovascular invasion (HR 0.62, 95% CI 0.21–1.86, *p =* 0.40) on bivariable analysis. There was no association of open approach with locoregional RFS (5-year locoregional RFS 92% versus 95%, log-rank *p* = 0.79 for both analyses). Due to insufficient events of interest, robust multivariable analysis of liver-RFS or locoregional RFS was not possible. 

### 3.4. Secondary Outcomes: Nodal Harvest and Margin Positivity

Of 259 patients with available data on lymph node harvests, the median nodal harvest in patients undergoing open procedures was 11 nodes (IQR 5–16 nodes), versus nine nodes (IQR 2–14) for patients undergoing laparoscopic procedures. When separated by operation type, there was no significant difference in mean nodal harvest for patients undergoing minimally invasive versus open distal pancreatectomies (eight versus 9.8 nodes, *p* = 0.16) or pancreaticoduodenectomies (17.2 versus 14.6 nodes, *p* = 0.28).

All patients had available data on operative margins, and there was no difference in the rate of margin positivity for patients undergoing minimally invasive versus open distal pancreatectomies (7/109 versus 6/75 patients, *p* = 0.77) or pancreaticoduodenectomies/total pancreatectomies (3/24 versus 6/61 patients, *p* = 0.71).

### 3.5. Secondary Outcomes: Perioperative Complications

In 159 patients undergoing proximal or distal pancreatectomy with available NSQIP data (*n* = 92 minimally invasive, *n* = 67 open), at 30 days postoperatively there were 16 (10%) incisional SSIs (*n* = 5 minimally invasive, *n* = 11 open) and 25 (16%) organ space SSIs (*n* = 10 minimally invasive, *n* = 15 open). Twenty-six (16%) patients required a blood transfusion in the 72 h following the start of the operation (*n* = 7 minimally invasive, *n* = 19 open). Thirty-four (21%) were re-admitted within 30 days (*n* = 18 minimally invasive, *n* = 16 open). Fourteen (9%) had an unplanned return to the operating room (*n* = 3 minimally invasive, *n* = 11 open). In 128 patients with procedure-targeted pancreatectomy data, there were 57 POPFs or biochemical leaks (45%, *n* = 37 minimally invasive, *n* = 20 open) and 18 crPOPFs (14%, *n* = 8 minimally invasive, *n* = 10 open).

After controlling for operation type (distal versus proximal pancreatectomy), a minimally invasive initial approach was associated with lower odds of incisional SSI (OR 0.29, 95% CI 0.09–0.96, *p* = 0.04), unplanned reoperation (OR 0.23, 95% CI 0.06–0.95, *p =* 0.04), need for perioperative blood transfusion (OR 0.19, 95% CI 0.07–0.51, *p* = 0.001), and crPOPF (OR 0.31, 95% CI 0.10–0.94, *p* = 0.04), but not with different odds of an organ space SSI (OR 0.53, 95% CI 0.20–1.42, *p* = 0.21), readmission within 30 days (OR 0.73, 95% CI 0.31–1.69, *p* = 0.46), or any POPF/biochemical leak (OR 0.92, 95% CI 0.42–2.02, *p* = 0.84).

## 4. Discussion

Novel drug, imaging, and radiotherapeutic technologies have advanced the care of patients with pancreatic neuroendocrine disease over the last decade, with improvements in disease recurrence, progression, and overall survival. Surgical approaches to the pancreas have also been augmented by increased enthusiasm for minimally invasive pancreatectomy, as well as the advent of robotic platforms [13,14]. The present results highlight the oncologic safety of minimally invasive approaches in this disease, as well as the equivalence or even superiority in perioperative outcomes with minimally invasive approaches.

The North American Neuro-Endocrine Tumor Society (NANETS) recently published expert-consensus guidelines on pancreatic neuroendocrine tumor surgical management, advocating for minimally invasive and gland-sparing resections when possible, limiting major pancreatic resections to aggressive, locally advanced, and high risk tumors [15]. The factors influencing choice in technique varies greatly depending upon tumor site, grade, extent of disease, and surgeon comfort, among other factors. Furthermore, treatment response to disease burden with newer chemotherapeutic approaches has changed the landscape of resections for locally advanced disease, as have increasingly aggressive vascular resections [16,17]. Given the variability in presentation of primary pancreatic neuroendocrine tumors—and their rarity—understanding trends in and benefits of minimally invasive approaches to resection remains challenging. While prospective data now exist supporting the efficacy and safety of laparoscopic distal pancreatectomy in tail lesions (LEOPARD Trial), which included benign and malignant pathologies, only one-third consisted of neuroendocrine disease [7].

Lacking the ability to feasibly study surgical approach in a prospective manner, retrospective evaluations must provide the support for safety, oncologic efficacy, and outcomes, in addition to illustrating trends over time in utilization. A number of single-center experiences have been published, but are limited by institutional bias and concentration of cases over a low number of surgeons [4,18,19,20,21]. Other single-center analyses of surgical approaches for PNETs have focused on specific types of minimally invasive approaches, such as enucleations, a technique that has been used in head tumors where otherwise a pancreaticoduodenectomy might be the alternative [22]. Multi-center collaboratives allow for large numbers of rare tumor resections to be evaluated, but also may bias results by comparing cohorts of patients treated at large, academic, tertiary centers [23,24,25,26,27,28].

Our regional approach combines several high-volume centers with varying patient populations, providing a more heterogeneous group of patients, surgeons, and care settings. Here, we contribute to the literature supporting the safety and oncologic efficacy of minimally invasive approaches to pancreatic neuroendocrine tumors, utilizing a large regional database of pancreatic resections. Our registry gives us a unique perspective on minimally invasive trends and efficacy in pancreatic surgery. The Greater Portland Pancreatic Cancer Registry was established in 2020 to aggregate the collective experience of major hospitals in the metro area. With the volume of neuroendocrine disease observed across our region, as well as a pioneering surgical culture that has been an early adopter and disseminator of minimally invasive techniques, this dataset offers a rich and progressive insight. Additionally, our results associating a minimally invasive approach with lower liver recurrence can be attributed to selection bias, as suggested by the lack of significance of operative approach after controlling for AJCC stage or lymphovascular invasion. Nonetheless, this important analysis suggests that a minimally invasive approach does not result in increased liver recurrence rates compared with open techniques, which is reassuring given that liver metastases drive survival in PNETs [29,30]. An analysis of this critically important oncologic endpoint has not previously been conducted within this population, and additional study is warranted.

Our data provide temporal insights into the changes in surgical approach for neuroendocrine tumors, both in head and tail resections. They also reflect a waning enthusiasm for enucleation, representing only 5% of our PNET resections, whereas early investigations of laparoscopic approaches to PNETs approached 20% [21]. This is consistent with published work documenting increased rates of postoperative pancreatic fistula when compared to head resections, and greater comfort with nonoperative management of small, low-grade PNETs [31,32].

Unsurprisingly, higher grade, more poorly differentiated tumors were more likely to be approached in an open fashion. Open approach was also utilized for higher stage, larger tumors, where nodal disease was more common. Pancreaticoduodenectomy was also more frequently performed in an open fashion, though nearly one-third were performed laparoscopically, a substantial number that reflects the adoption of this technique for neuroendocrine disease and the regional experience with complex minimally invasive approaches. The low rates of R1 resections in both groups, as well as the excellent recurrence free- and disease-specific survival across approaches indicates the appropriateness of minimally invasive resections when selected by surgeons in our registry. The persistence of this finding, especially in a more complicated minimally invasive procedure, is significant, as pancreaticoduodenectomy supports the noninferiority of this technique when appropriate. The trend towards improved recurrence-free survival in minimally invasive distal pancreatectomies is also encouraging, but likely reflects preoperative decision making on approach rather than improved technique.

By limiting our analysis to resections of primary tumors, we attempted to align our cohorts by removing resections, including pancreatectomy and hepatectomy for metastasectomy, procedures that are far more likely to be performed via open approach. Our analysis has attempted to provide a robust yet well-matched comparison between these approaches, but suffers from the limitations inherent in retrospectively comparing technical approaches that change over time. As such, our study is limited by selection bias both due to time of diagnosis, and selection bias toward performing minimally invasive techniques for smaller tumors without adjacency to major vessels. Additionally, our study is limited by a small sample size and low statistical power, largely due to the rarity of this population. Consequently, while we considered propensity-matching methodologies, we felt that the loss in power from patients excluded during matching would outweigh the statistical benefits given the low events of interest. Additionally, for oncologic outcomes year of diagnosis would be a statistically but not clinically important component of a propensity matching approach, given the era-dependent utilization of minimally invasive techniques. An additional limitation of the study is that there was no grade III well-differentiated PNETs identified in our study, which is a new classification in the WHO 2017 guidelines. This may in part reflect incomplete staging or limitations of the NAACCR-formatted data. Large international investigations into resected pancreatic neuroendocrine tumors and carcinomas also suggests that the 2017 addition of this category may be of more utility for medical management of these tumors, as they behave similarly to neuroendocrine carcinomas [33].

## 5. Conclusions

In this large, regional, multi-center retrospective analysis of surgical approach for primary pancreatic neuroendocrine tumors, we have provided additional support to the emerging work on the safety, utility, and oncologic efficacy of minimally invasive approaches for pancreatic surgery in neuroendocrine disease. This approach allows for a reduction in the risks associated with pancreatic surgery and increases access to potentially curative surgery in a larger group of patients.

## Figures and Tables

**Table 1:** Clinicopathologic characteristics of patients undergoing resection of pancreatic neuroendocrine tumors.

Variable	Open (N = 139); N (%)	Minimally Invasive (N = 143); N (%)	All Patients (N = 282); N (%)	*p* Value
Age, Years; Median (IQR)	56 (46–67)	61 (50–69)	59 (48–68)	0.21
Sex				0.48
Female	72 (51.8)	68 (47.6)	140 (49.6)	
Male	67 (48.2)	75 (52.4)	142 (50.4)	
Operation Performed				<0.001
Distal pancreatectomy/RAMPS	75 (53.9)	109 (76.2)	184 (65.2)	
Enucleation	3 (2.2)	9 (6.3)	12 (4.3)	
Enucleation + lymphadenectomy	0 (0)	1 (0.7)	1 (0.4)	
Total pancreatectomy	7 (5.0)	1 (0.7)	8 (2.8)	
Pancreaticoduodenectomy	54 (38.8)	23 (16.1)	77 (27.3)	
Tumor Location				<0.001
Body/tail	57 (41.0)	90 (62.9)	147 (52.1)	
Head/neck	55 (39.6)	25 (17.5)	80 (28.4)	
Overlapping/not specified	27 (19.4)	28 (19.6)	55 (19.5)	
Functional Tumor	8 (5.8)	2 (1.4)	10 (3.5)	0.048
AJCC Stage				0.008
I	27 (19.4)	53 (37.1)	80 (28.4)	
II	65 (46.8)	58 (40.6)	123 (43.6)	
III	37 (26.6)	26 (18.2)	63 (22.3)	
Incompletely staged	10 (7.2)	6 (4.2)	16 (5.7)	
Tumor Size, cm; Median (IQR)	3.3 (1.7–5.3)	2.1 (1.2–3.0)	2.5 (1.5–4.0)	<0.001
Lymphovascular Invasion	27 (19.4)	23 (16.1)	50 (17.7)	0.53
Nodal Status				0.10
pN0	92 (66.2)	111 (77.6)	203 (72.0)	
pN1	37 (26.6)	26 (18.2)	63 (22.3)	
pNx	10 (7.2)	6 (4.2)	16 (5.7)	
Differentiation				0.04
Poorly	9 (6.5)	5 (3.5)	14 (5.0)	
Well	105 (75.5)	125 (87.4)	230 (81.6)	
Not specified	25 (18.0)	13 (9.1)	38 (13.5)	
Tumor Grade				0.07
I	77 (55.4)	96 (67.1)	173 (61.3)	
II	28 (20.1)	29 (20.3)	57 (20.2)	
III	9 (6.5)	5 (3.5)	14 (5.0)	
Not specified	25 (18.0)	13 (9.1)	38 (13.5)	
Surgical Margins				0.77
R1	12 (8.6)	10 (7.0)	22 (7.8)	
R0	127 (91.4)	133 (93.0)	260 (92.2)	
BMI *, kg/m^2^; Median (IQR)	29.5 (25.1–32.2)	30.0 (25.1–33.6)	29.8 (25.1–33.2)	0.46
ASA Class *				0.87
1–2	23 (34.3)	34 (37.0)	57 (35.8)	
3–4	44 (65.7)	58 (63.0)	102 (64.2)	
NSQIP-defined Comorbidities *				0.44
0	24 (35.8)	33 (35.9)	57 (35.8)	
1–2	40 (59.7)	50 (54.3)	90 (56.6)	
3–5	3 (4.5)	9 (9.8)	12 (7.5)	

* For 159 patients with available NSQIP data (*n* = 92 minimally invasive, *n* = 67 open). Abbreviations: IQR = interquartile range; AI/AN = American Indian/Alaskan Native; RAMPS = radical antegrade modular pancreatosplenectomy; AJCC = American Joint Commission on Cancer; BMI = body mass index; ASA= American Society of Anesthesiologists; NSQIP = National Surgical Quality Improvement Project.

**Table 2:** Multivariable analysis of 5-year recurrence free survival in patients with pancreatic neuroendocrine tumor.

Variable	Univariable HR (95% CI)	*p*	Multivariable HR (95% CI)	*p*
Age (per year)	0.99 (0.97–1.01)	0.33	-	-
Male sex	0.95 (0.47–1.90)	0.88	-	-
LVI absent	Referent	-	-	-
LVI present	5.19 (2.18–12.34)	<0.001 *	-	-
T1/T2	Referent	-	Referent	-
T3/T4	6.42 (2.82–14.58)	<0.001	2.78 (1.07–7.23)	0.04
Node positive	3.37 (1.68–6.77)	0.001 *	-	-
Grade 3	Referent	-	Referent	-
Grade 2	0.17 (0.07–0.43)	<0.001	0.20 (0.07–0.54)	0.002
Grade 1	0.04 (0.01–0.10)	<0.001	0.07 (0.02–0.22)	<0.001
R1 margin	3.33 (1.44–7.71)	0.005	2.43 (0.96–6.15)	0.06
Minimally invasive approach	0.58 (0.28–1.19)	0.14 *	-	-

* Removed from final model following single backward elimination. Abbreviations: HR = hazard ratio; CI = confidence interval; LVI = lymphovascular invasion.

**Table 3:** Univariable analysis of 5-year disease-specific survival in patients with pancreatic neuroendocrine tumor.

Variable	HR (95% CI)	*p*
Age (per year)	1.06 (1.00–1.13)	0.049
Male sex	0.59 (0.14–2.47)	0.47
LVI absent	Referent	-
LVI present	14.68 (1.64–131.33)	0.02
T1/T2	Referent	-
T3/T4	13.14 (1.58–109.19)	0.02
Node positive	3.49 (0.87–13.98)	0.08
Grade 3	Referent	-
Grade 2	0.04 (0.01–0.37)	0.004
Grade 1	0.02 (0.01–0.13)	<0.001
R1 margin	1.49 (0.18–12.10)	0.71
Minimally invasive approach	0.61 (0.18–2.04)	0.42

Abbreviations: HR = hazard ratio; CI = confidence interval; LVI = lymphovascular invasion.

## Data Availability

Data are available from the corresponding author upon reasonable request and with approval of the OHSU IRB.
